# Lecturers’ experiences of online learning communication in Indonesia during the COVID-19 pandemic: A phenomenological study

**DOI:** 10.12688/f1000research.141212.1

**Published:** 2024-03-08

**Authors:** Anter Venus, Lukman Saleh Waluyo, Fitria Ayuningtyas, Kusumajanti Kusumajanti

**Affiliations:** 1 Communication Science, Universitas Pembangunan Nasional Veteran Jakarta, Jakarta, Special Capital Region of Jakarta, 12450, Indonesia; 2 School of Management and Marketing, Curtin Business School, Curtin University, Perth, Western Australia, Australia

**Keywords:** online learning, communication, phenomenology, qualitative, Indonesia, lecturer, pandemic, digital

## Abstract

**Background:**

Indonesia is currently implementing educational reforms to enhance university teaching and learning. The COVID-19 pandemic has significantly affected Indonesian universities, requiring lecturers to implement various changes. The phenomenon of online learning communication at various universities in Indonesia is critical considering today’s increasingly fierce competition due to digital disruption coupled with the impact of the pandemic. Therefore, lecturers must implement appropriate methods to conduct online learning communication effectively.

**Methods:**

This qualitative phenomenological study was designed to explore lecturers’ understanding of the implementation of changes in online learning communication at Indonesian universities. In-depth interviews and field observations were conducted with lecturers who were efficient in online learning using a specific measurement standard. The research participants were identified using purposive sampling with a snowball approach. The study comprised 10 lecturers from UPN Veteran Jakarta who were experienced and efficiently delivered online lectures.

**Results:**

The results provide a review of factors identified by the phenomenological and qualitative study that contribute to online learning communication, such as chemistry, lecturer preparation, and learning materials. The results of this study revealed several themes. Each section centers on the major themes that emerged from the datasets.

**Conclusion:**

Our findings aid in a better understanding of the benefits and constraints of using online learning communication in the classroom. These findings are crucial for university lecturers developing methods for enhancing the implementation of online learning communication. Moreover, the findings revealed that lecturers should engage in and create trusting interactions with their students. Providing technical support to keep both teachers and students engaged can be useful. More crucially, multimedia support and learning systems are essential for properly addressing lecturers’ tasks and learning materials. This study improves the understanding of online learning communications implemented by lecturers.

## Introduction

The expansion of online learning is rapidly transforming higher education, with an increasing number of colleges offering degree programs in online and remote learning modes worldwide. Traditional “correspondence” study has transitioned into online learning as a result of the digital communication breakthroughs of the 21st century, with many more universities, both urban and regional, providing programs that may entirely be pursued online. ^1^

The COVID-19 pandemic has significantly impacted almost all aspects of our lives, including the Indonesian education sector. This has a significant effect on various learning activities. During the pandemic, the government-imposed restrictions on interactions and activities outside the home to limit the spread of the coronavirus infection, which hampered conventional learning methods.

Several measures were implemented to ensure that educational activities continued despite the lack of face-to-face instruction. In particular, students and lecturers in higher education were unable to meet in the classroom; therefore, they adapted to changing circumstances, and devised new tactics such as implementing online learning. ^2^

Communication, particularly in the educational sector, should be researched, because communication between instructors and students has the potential to promote learning and create a positive environment. ^3^

Technology plays an important role in the continuity of learning through non-conventional media such as online media. Online learning is considered a solution that could connect teaching and learning activities during the pandemic. Most educational institutions have shifted their learning activities to the online mode. Therefore, online learning communication is an important aspect that must be investigated by stakeholders in the field of education including lecturers, students, schools, and universities. The current rapid development of technology and communication has had a significant impact on the changes that occur in society, including teaching and learning processes. Therefore, online learning communication by lecturers during the pandemic is an important research subject. The expected findings of this research reveal how the phenomenon of online learning communication in suitable public universities can encourage the development of science, technology, and social sciences.

The subject of online learning was selected because researchers believe that there were several new and unique aspects of online learning implemented during the COVID-19 pandemic that could not be conducted during face-to-face learning.

One of the policies implemented by the Indonesian government to prevent the spread of COVID-19 was the implementation of Large-Scale Social Restrictions (PSBB), one of which was to close schools. Over a period of almost three years since the outbreak of the pandemic, several changes have occurred in the world of education and have been experienced by students since the implementation of complete online learning. These changes are evident from various aspects of online learning activities, including the media used, interactions that occur, materials provided, and methods used. These changes have led to various communication experiences for students. Such a novel experience certainly has its own value or meaning for individuals who experience it first-hand. The more important the value of an experience, the more memorable it will be.

Higher education is a more complex level of education than a school education. Higher education aims to develop graduates with high professional and academic abilities to develop or create science, technology, and arts to improve human welfare and national development. Therefore, scholars have focused on examining students’ online learning communication experiences during the COVID-19 pandemic.

As online learning is ongoing due to the COVID-19 pandemic, this topic is relevant and vital for publication. It is essential to evaluate the problems pertaining to online learning communication experiences and online learning policies set by the government to prevent or minimize the spread of COVID-19 in Indonesia. It is expected that the results can be used to improve the quality of online learning in Indonesia.

### Literature review

Rapid advances in science and technology have had a tremendous impact on social habits in recent years and have resulted in significant changes in the production, distribution, and communication of information. Consequently, the rate of knowledge expansion has accelerated, necessitating a constant update of current information. Simultaneously, the need for people to constantly analyze information and remain up to date with daily advancements is a top priority. Consequently, the use of information and communication technology (ICT) and the internet in education has increased, culminating in the proliferation of online environments. ^4^

In online learning communication, from the students’ viewpoint, the lecturer needs to communicate with the students using informal channels (instant messaging, online chat groups, voice calls, and video calls) parallel alongside formal channels. ^3^ Students and lecturers face several challenges in online learning communication, including a lack of technological proficiency, increased Internet expenses, and erratic communication and socialization between students. ^5^

Economic situations, anxiety during online learning, the risk of user data security, finding suitable online learning media, and expectations are some of the problems students face while studying online. ^2^

Out of a broad range of learner characteristics, the factors that enable Generation Z students to succeed in dedicated online learning communication are the learners’ home institutions, which substantially impacted their preference for instructional delivery modality. According to students, the best aspects of a dedicated online course are a flexible timetable and quick access; however, they also want more real-time interactions with teachers and classmates, multimedia resources, and various applications (apps). According to students, metacognitive and thinking skills are essential for success in dedicated online courses. ^6^

The ability to create a sense of presence for improving learning outcomes is an important aspect of online learning. Students should be provided with immediate feedback, meaningful learning experiences, and opportunities to form relationships with classmates and professors to improve their presence. ^7^

Student happiness in online learning was significantly influenced by social presence, fueled by tool engagement. Moreover, in online learning, sex differences affect the links between tool interactions and social presence. ^8^

Students revealed the various challenges they faced when using information technology (IT) platform applications for online learning. Work and information overload received from instructors via online learning platforms, technological hurdles connected to unaffordability of online learning support facilities, and mental health challenges related to stress and anxiety are examples of such obstacles faced by students. ^9^

Online learning communication presents several technical, educational, and social challenges. Technical challenges are primarily related to the unreliability of internet connections and the lack of electronic equipment required by students. Pedagogical challenges include a lack of digital skills for teachers and learners, a lack of structured content, an abundance of online resources, learner interaction, lack of motivation, and teachers’ social and cognitive skills. Regarding the lack of presence, building content together creates meaning through continuous communication within the research community.

Social factors are mostly due to the lack of human engagement between instructors and learners, lack of private physical spaces at home for students to attend lessons, and lack of support from parents who simultaneously work remotely in the same spaces. ^10^

Time restrictions, low technological skills, inadequate infrastructure, a lack of institutional strategies and support, and negative attitudes of those engaged are some of the major challenges in the design and implementation of online learning communication in education. Improved educator skills, incentives, and rewards for the time spent developing and delivering online information, improved institutional strategies, support, and a positive attitude among all those involved in the development and delivery of online content are some of the solutions to these problems. ^11^

The most significant component for both teachers and students in their online teaching and learning experiences was holding students to high standards of performance, academic honesty, and professional ethics. The faculty requires a well-designed online classroom with engaged students who complete their work on time, and students require an online instructor who is organized and communicative in the online classroom. ^12^

In comparison with offline classrooms, student participation in online learning may be vague and difficult to understand. Without face-to-face interactions, instructors may be unable to measure student involvement accurately or adjust accordingly. ^13^

Face-to-face learning was rated higher than online learning in terms of social presence, social engagement, and satisfaction. However, no statistically significant differences in learning preferences were found among students. Some students preferred online learning because it provided them with opportunities to be creative using computer technology. ^14^

Students’ engagement plays a key role in online learning. Student engagement is a four-dimensional construct with behavioral, cognitive, affective, and agentic components. ^15^ Naji *et al.* ^16^ recently conducted research on engineering students to determine the factors influencing their willingness to learn online during the COVID-19 pandemic. They discovered that four variables influenced students’ preparedness for online learning: 1) initial preparedness and motivation, 2) self-efficacious attitudes toward online learning, 3) self-directed online learning, and 4) support for online learning. Furthermore, social media platforms such as Facebook and Instagram can be used for online learning. ^17^

Online learning is a new experience that allows students to study easily, without having to travel to campus. However, challenges persist in online learning. Due to the lack of effective network access, communication and directions in lectures are unclear. Student participation in online courses is hampered by the limited budget for Internet subscriptions. Student focus suffers as a result of excessive workload. The government should ensure that all regions have sufficiently stable Internet network connections and universities must provide students with subsidized Internet subscriptions to eliminate unaffordability. Consequently, lecture activities can proceed easily as planned. ^18^

### Research questions

Understanding and determining the factors of online learning communication from a lecturer’s viewpoint are essential for promoting successful online learning communication.

Although studies assessing strategies to improve online learning communication have been conducted, none have been conducted from lecturers’ perspectives. Therefore, a review of the narrative and qualitative studies is required.

We reviewed observational and qualitative studies to identify the factors that lead to successful online learning communication from lecturers’ perspectives. Qualitative studies are necessary because of their methodology (the approach to answering questions about experience, meaning, and perspective), which is useful for addressing implementation issues.

Furthermore, no studies have addressed this subject in Indonesian culture. Therefore, this qualitative study was conducted to explore the concept of online learning communication in Indonesian society.

This study explores lecturers’ experiences with online learning communication. Lecturers were asked to describe and discuss their perspectives regarding their experiences and views, the challenges they faced during online learning communication, and the types of support they required. The research questions that guided this study were as follows:

1. What are lecturers’ experiences when implementing online learning communication?
2. How do lecturers provide meaning to online learning communication?
3. What are the challenges that lecturers face during online learning?

## Methods

### Ethics and consent

All participants provided written informed consent before participation. Because they participated voluntarily, they were free to leave the interviews at any time. All the respondents provided informed consent. Subsequently, written consent was obtained to share the anonymized data. This research has been ethically approved on April 30th, 2021 by the research ethic commission of UPN Veteran Jakarta (No: 346/VIII/2023/KEPK).

### Study design

This study employed a qualitative research method to analyze the data from the research results. This method allows holistic interpretation and explanation of a phenomenon regarding online learning communication experiences using words, without having to rely on quantitative data.

Qualitative research conducted aimed to determine and understand this phenomenon in depth. Data analysis was performed inductively. The processed data emphasized the depth (quality) of the data obtained more than the amount (quantity) of data.

Using qualitative research methods [ 1] allowed for the analysis of words or sentences uttered and the situation or conditions experienced by the informants in this study from their perspective. This approach focuses on using analyses and existing theories or concepts as supporting materials for creating a theory. The results of the study included interactions with the participants and observed behaviors. Data were collected from notes, memos, interview scripts, and other supporting documents.

To understand lecturers’ experiences in online learning communication, this study used a phenomenological [ 2] approach to reflect on and describe their experiences through interactions with the interviewer. ^19^ The purpose was to accurately portray these lecturers to describe their observations and discover new meanings.

The phenomenological method used in this study was Heidegger’s hermeneutic phenomenology, which is oriented toward searching for the meaning of experience for the participants. Heidegger’s phenomenology is more flexible in identifying and validating research results. Heidegger developed a phenomenological method that allows for the inclusion of prior experience and presuppositions or preconceived knowledge from informants in research data. In this way, validation is no longer required because during the research process, participants and researchers are intensely involved in the process of sharing experiences. ^20^

### Participants and settings

This research was conducted at a public university with approximately 10,000 students enrolled. This location was selected because it was possible to acquire access and cooperation between the administration and faculty members. Almost 500 lecturers from various backgrounds constituted university personnel. None of the lecturers were foreigners. The official language of instruction is Indonesian, and classroom contact hours are two hours and 30 minutes.

All university lecturers were invited to participate in the study ( Table 1). Consequently, all participants were volunteers. The study sample comprised 10 lecturers from UPN Veteran Jakarta, who were experienced and considered excellent in delivering online lectures. The data were collected between June 2021 and April 2022. This study used nonprobability sampling with a snowball sampling technique. Snowball sampling was used to identify key informants who had significant information. Using this approach, several potential respondents were contacted and asked whether they knew others fitting the research criteria. The initial contact helped obtain recommendations from other respondents.

**Table 1.** Overview of participating lecturers.

No	Study area
1	Health
2	Environment
3	Journalism
4	Public Relations
5	Law
6	Economy
7	Computer
8	Politics
9	International Relations
10	Engineering

### Data collection

This study used in-depth interviews to obtain information and data by asking the informants several questions ^33^ about the phenomenon of online learning communication. This strategy was selected for its adaptability and capacity to boost valuable intelligence among people while expanding the understanding of the concepts being studied.

The in-depth interviews aimed to explore participants’ in-depth experiences during online learning communication. The investigator informed the participants that they could withdraw at any time during the study and that they had the option to not respond to any of the questions.

During the discussions, the participants were encouraged to express their opinions and experiences openly. Probing was used throughout the interviews to further develop lecturer responses and applicable samples. Each interview was audio recorded and converted into verbatim transcriptions to present the findings in narrative form. Moreover, handwritten notes were prepared to track valuable insights that could enrich the findings. To validate the results, each party was given an interview summary for a final review. The interviews took place at the university and required approximately 30 minutes to answer the research questions, and the participants answered five interview questions. The locations for group discussions were selected by the interviewees ( *e.g.*, workplace and online). At each meeting, the interviewer (the corresponding author) clarified the ponder’s goals. An audio recorder was used for data collection.

### Data analysis

Qualitative data analysis was performed to develop the complex meanings that emerged from the raw data. Data analysis was performed by experts on the research team (dependability). The interviews were transcribed, ^31^^,^^32^ and the data were analyzed through a phenomenological approach using survey questions as a guide. The study was designed using the following four levels: horizontalization, presentation of significant statements, analysis of themes (thematization), and phenomenological reduction (crystallization). The data analysis was performed both manually and using the software NVivo 14.

In the horizontal stage, the researcher describes and presents all data obtained from the informants, as they are placed and treated equally. Horizontalization is the stage of presenting data as a whole, where all data receive the same place and treatment.

The second stage involved presenting significant statements. During this stage, the researcher reduced the research data or sorted out the statements of the informants disclosed during the interview that were considered relevant to the research question. The researchers marked the relevant statements to distinguish them from irrelevant statements and flattened them (invariant ons/units of meaning of phenomena).

The third stage was a thematic analysis. In this case, themes were defined as a broad categorization of feelings, thoughts, and meanings that represented the essence of each participant’s experience. The theme in this context is important because it made it easier for researchers to map out the experiences of the participants and focus on examining the specific experiences of participants that have similarities with other participants.

The essence is the crystallization of several short and dense themes. ^21^ In the essence of the experience stage, the researcher attempts to determine the essence of the research, which is the result of the crystallization of several short and concise themes, by rereading the identified themes, combining them, determining the most general statements of these themes, and covering all the themes that have been identified.

All the data required to explore these important themes were obtained from direct interviews as first-hand sources of information. Thus, the scientific conceptualization of the participant experience can be conducted validly without bias or distortion. In accordance with the process of analyzing Heidegger’s hermeneutic phenomenology, which emphasizes previous knowledge about the phenomenon under study, important categories or themes will first be presented and then juxtaposed with the themes that emerge from the interpretation process conducted by the research. ^22^

### Trustworthiness

Tracy’s ^23^ eight-dimensional model for quality model was used to establish rigor in qualitative research: (a) worthy topic, (b) rich rigor, (c) sincerity, (d) credibility, (e) resonance, (f) major contributions, (g) ethics, and (h) meaningful coherence. In addition, researchers have used various tactics to improve the credibility, transferability, dependability, and confirmability of their results. Prior to data analysis, participants were permitted to double-check their responses through member checking. ^24^ Following completion of the data analysis, each participant examined the interpreted findings for data validation and verification. Second, the researchers thoroughly described the data collection and analysis techniques, cross-checked emergent themes to eliminate repetition and data overlap before presenting them as narrative paragraphs and assessed the study protocol to allow for research replication. ^25^ Finally, researchers aware of the research setting used journal logs to reflect on their views while completing the study to avoid bias. Consequently, they were able to eliminate subjectivity, retain objectivity, and maintain neutrality when collecting and analyzing data.

## Results

The results of this study revealed several themes. The first theme answers the first two interview questions and summarizes the lecturers’ experiences regarding the most challenging aspects of implementing online learning communication.

Thereafter, the remaining interview questions were addressed, and the superiority of digital learning was reported. Each section centers on the major themes that emerged from the datasets. Finally, the discussion addresses lecturers’ perceptions of the most significant requirements to implement successful online learning communication at universities and draws a connection between the findings of this study and extant studies.

### Lack of engagement

According to the participants, lecturers experienced several challenges in online learning communication. The most significant challenge for the lecturers is their lack of engagement in the learning process. In particular, the lack of interaction, chemistry, personal touch, and gestures were significant concerns for the lecturers.

### Interaction

Seven lecturers reported interactions as a challenge in online learning communication. In support of this argument, a participant reported, “The ideal communication in online learning occurs when there is interaction between lecturers and students. If it occurs only in one direction, it is not effective, because if the student continuously focuses on the monitor without being allowed to interact, it could tire their eyes.”

### Chemistry

Lecturers have experienced chemistry as a challenge in online communication. In support of this argument, a participant explained, “The most noticeable difference is in terms of chemistry and feedback from students. Even in online learning communications, one can receive direct feedback, however, in my opinion, receiving feedback is not similar to chemistry. Not speaking directly makes you feel less comfortable; therefore, you tend to get bored, and those you talk to also tend to get bored easily. This is because they speak in front of a laptop and do not directly interact with people; hence, there is a problem owing to the lack of human communication.”

### Personal touch

Personal touch was a significant concern for the lecturers. Respondents argued that personal touch was a challenge when implementing online learning communication.

For example, a participant reported different experiences: “There is no conversation, there is no eye contact, or physical and mental contact. Although I can see their faces, my personal bonds are missing. Although the camera features are on, they are different from real classrooms.”

### Gesture

The lecturers experienced that understanding gestures in online learning communication that account for students’ learning styles was a challenging task.

Speaking about this issue, a participant mentioned, “I am unable to assess accurately during online learning; it means that I am not sure about the student’s understanding of the lecture. In the offline classes, this can be understood from their gestures. Although they turn on the camera, much delay occurs during the learning process; therefore, we cannot be sure whether they have understood the material taught and their experiences during the lecture.”

### Technical problems: connection, device, camera, microphone, quota, and noise

It is clear from lecturers’ experiences that various technical problems occur during online learning communication. In support of this argument, a participant stated, “While teaching online, some students have problems with cameras or microphones. It is difficult to ascertain whether it is simply a cry or whether there are defects in laptop devices. Therefore, I sometimes go back and forth to remind them to turn the camera on. The drawbacks are almost the same, perhaps network problems, because many of them do not come on camera when called; the reason is because the network is unstable. Furthermore, if they do not turn on the camera, we cannot determine the expression of the students at all or their interests during the lecture. Therefore, I prefer them to have cameras on them. Weather also affects they cannot be on camera. Regarding devices, several students do not have adequate laptops, computers, or any equipment at all and therefore use cell phones.”

The lecturers stated that online communication was ineffective. According to a participant, “I believe the effectiveness is lower than during face-to-face learning. The online medium can have several elements that hinder its effectiveness, for example, technical connections, device readiness, and existing features or facilities in the application.”

Similarly, a participant indicated, “Devices and technology that do not support, and cause internet network connection disturbances affect the mood of students as well as the mood of the lecturers. During a lecture, suddenly if the internet connection is interrupted, we forget what we are talking about. Restoring network connections is also time consuming. This affects the students’ moods and their ability to continue with the same interest.” These lecturers argued that they could not effectively implement online learning communication because of the noise. Speaking about the importance of noise free environment in online learning communication, a participant stated, “For example, during lectures, students often make presentations, and they are unable to mute their surroundings. This could disturb the class.” A participant further explained, “The more disturbing aspect is when they do not use a virtual background and family members can be seen going back and forth in the background. In one instance, the parent could be seen with towels in the background. This may be due to student negligence or ignorance. Additionally, sometimes when I ask a question, they turn on the microphone and the sound varies, possibly because they commute. The sound was noisy and disturbing; hence, I had to transfer the question to another student.” These experiences demonstrate their need for an environment where they feel secure in expressing themselves and have their voices heard in online learning communication. A participant commented, “Honestly I do not ask my students to turn on the camera, except in cases of direct interaction, as students say that turning on the camera consumes a large quota of internet.”

### Hybrid learning based on the subject

Several respondents had different views on whether communication learning should be conducted online or face-to-face. However, 40% of the participants were of the opinion that the method of learning implementation must be adjusted according to the subject being taught.

For example, as conveyed by a participant, “In my opinion, students continue to prefer online learning because they are comfortable. However, certain courses are difficult to teach completely online. For example, courses on quantitative research method courses. These courses require full supervision from lecturers to ensure that students understand them thoroughly. If it is taught completely online, it will be somewhat difficult. However, complete online learning may not be problematic for other courses. Thus, it depends on the course and learning outcomes you aim. Therefore, we cannot equate one course with another, because each course has its own approach. Maybe several activities may be combined for online learning instead of being taught in a Zoom meeting or Google Meet. The courses can be conducted both online and offline, so that some activities can be conducted online, and the students can gather together for the offline activities.”

A participant had the same view, “Perhaps there are some courses that must be attended offline. For example, courses are offered by the Faculty of Engineering, Faculty of Medicine, or Faculty of Health Sciences. However, some humanities courses can be conducted completely online. In some countries, the number of offline classrooms has decreased, therefore, those that can be conducted online are expected to continue. Online learning communication is beneficial for all parties, not only for lecturers but also for students who may be living far from campus; however, they can continue to study online. Moreover, they do not have to search for boarding houses or temporary accommodation while studying at the UPN.”

A participant stated, “I believe in a more hybrid direction, hence, it is not 100% online or 100% offline. Although it is true that online learning communication benefits lecturers in terms of expenses such as saving transportation expenses, it also reduces teaching satisfaction because interactions with students are mediated. Online learning cannot replace offline learning completely. Some lessons should be conducted online, while others should be conducted offline. For example, general-knowledge classes can be conducted online. However, in the context of research or skill practicing a skill, it is more effective if conducted offline. Hence, I believe that the hybrid format is interesting to achieve the ideal format in learning for better education.”

### Maximizing Efficiency of Online Learning Communication

According to lecturers, several methods can be employed to maximize the efficiency of online learning communication. First, lecturers are willing to upgrade themselves to become acquainted with current online learning communication technology.

A participant stated that, “Lecturers and students both have much knowledge of learning technologies, such as Zoom, Google Meet and other platforms. We have to become used to mastering the technology used to support online learning communication processes. Several types of platforms can be used; however, not all lecturers understand how to use them, hence, they must be trained. If a lecturer is aged 45 years or younger, it may be easy to adapt. However, if the age is above 45 years, it is difficult to train them as they need assistance, even in creating a conference link or sharing their screen. How do they aim to create learning innovations with the so many types of innovation provided on the Internet? Lecturers should apply this new development that must be applied in the future. Therefore, they are willing to learn and change. Commitment is the most important because as lecturers, we must adapt to changes to provide the best for our students.”

Similar views were shared by a participant. “Lecturers as educators must be able to follow the ongoing learning trends that are occurring worldwide. They must remain updated to ensure that they are not left behind. They have been provided complete information using videos and manual books and are also assisted by the e-learning team in each faculty so that they have no difficulty in using online learning communication.”

Moreover, the delivery of learning materials is important. Lecturers believe that several methods may be used to maximize the deliverability of learning materials. A participant believes that “the point is how to engage students in the learning process. I attempt to make them more focused on the choice of words and intonation of the voice, which is firmer and heavier. This makes them more attentive to what I say than when I speak to them jokingly or casually and with normal intonation. I used diction that is firmer and straightforward because maybe they are studying while lying down or doing something else. We must be more dominant than in other activities. I stand even up when delivering the material is delivered. I believe that it is useful for me because it stimulates my brain. Moreover, for students, it offers more varied body and hand movements than when I teach while sitting. Additionally, while standing, I can stretch and draw attention.” The importance of diction was also stated by a participant, “The second step is to select words. Good diction will draw their attention. We teach verbally, with the help of PowerPoints, photographs, and words. This is more than simply reading the material.”

### Learning duration

Participants differed in their views on the best duration for online and offline learning. According to a participant, based on my experience, if we use the same duration as offline learning, it will not be effective because the student will experience visual fatigue. If we speak normally, it is usually only effective for one hour, at the most. Therefore, our problem is how to use the two hours effectively and keep students engaged. Hence, I have adopted an approach in which I post all types of explanation materials first. I asked my students to read/watch it according to their convenience so that during class, their doubts and questions could be clarified. Later, we can assign them to a case study.” Other participants had similar views. A participant believes that it is important to provide breaks during the learning process. “I usually stop every 45 minutes because student focus is limited. If lectures are prolonged, students do not concentrate. Therefore, I provide a break of 10 min and then continue again.” Another participant stated, “In other universities, such as UNDIP (Diponegoro University), they rarely conduct synchronous learning. The maximum synchronous learning was only half of the course. However, the maximum duration should not be more than 30 min because students’ focus on online learning is limited. After 20 min, people typically lose interest. Moreover, students were happy that way as they did not have to be glued to Google Meet or Zoom all day. At least 20-30 minutes is sufficient to talk about issues that need to be discussed or explained further.”

### The use of multimedia

Online learning communications provide features and facilities that cannot be found in offline learning such as video conferencing, animation, recorded videos, visual graphics, forums, quizzes, and attendance. Most lecturers have specific views on this topic. A participant believes that “in online learning communication, we can use many forms of media, such as video and animation.” Another participant believes that online learning communication that has been running at the UPN is useful because it not only provides learning material but also allows interaction through conferences or videos. Online learning is a model of futuristic learning. In the future, campuses may not require several rooms, because they can provide learning anytime and anywhere. Moreover, online learning videos can facilitate knowledge transfer from lecturers to students in a unique manner that may not be possible in offline classrooms.” A participant said, “I rarely conduct lectures during online classes.” In my experience, this method was not attractive to my students. Therefore, I usually assigned a video lesson to students and asked them to watch it before the next class. Thus, when the class begins, it is time for discussion, not for listening to my lectures.” According to a participant, “I told them that in my class I would not be lecturing on the material. I will record the learning materials and share the YouTube links. This recording is a significant effort, as my recordings are usually between seven and 10 minutes long. During class, we elaborated on this. Therefore, I do not expect that I will explain the material in class as it has been provided through video recordings, and I have uploaded all the references to the LMS.” Similarly, another participant stated, “Another option is to reduce the duration of lecture by providing video tutorials. Thus, we can provide interactive videos with sufficiently interesting graphics to keep the users absorbed.”

### Learning method

Lecturers have highlighted dyadic communication as an effective method of online learning. Therefore, methods that support two-way engagement such as presentations, experiments, quizzes, projects, case studies, and forums should be employed. As a participant stated in the interview, “We should not lecture anymore. Lecturers only facilitate students in the direction of their learning using a dialogue model and not one-way communication. Thus, we continue to have several challenges, as we are not directed there.”

Another participant supports similar methods of online learning communication. “We must provide incentive to students to encourage them to ask questions. I began my lecture with a quiz, as the video material had already been provided to them. There are several questions in the quiz, those who ask them are given a score, and they must be transparent. This process becomes easy with the use of learning apps. We created a discussion forum for those who asked questions. They were assessed and provided with feedback or a score.” A participant believes, “We can interact with students by providing them problems such as a case study. For example, I teach health service management and suggest, “You become a director at a hospital or the head of a *puskesmas.* How would you position yourself? What would you do?” Therefore, a discussion was initiated. However, it is difficult to identify relevant cases, as it requires practical experience.” Another participant stated that another method that can be used in online learning communication is to increase students’ activities at home, a type of pre- or post-activity before or after teaching. For example, provide them with a topic of discussion before lectures that can later be discussed in class. Alternatively, after lectures, they can be asked to read about or discuss a topic that they will bring up the following week. In online learning communication, everything is integrated into a single information system; thus, pre pre-activity or post-activity is integrated into the information system, making it easier for lecturers to manage.”

## Discussion

This study identified student engagement as the most significant aspect of implementing online learning communication with respect to lecturers’ experiences of online learning communication in Indonesia during the COVID-19 pandemic. The lecturers discovered that student interaction primarily centered on the need to improve their online learning communication practices and that online engagement was highly valued by students for online learning communication. Students identified small-group interactions as the most important aspect of communication; however, non-participation by some members was cited as a major issue. ^26^

More specifically, lecturers face challenges such as technical problems, duration, learning methods, and the use of multimedia. These findings align with previous research on lecturers in online learning communication.

Some lecturers believe that using online learning communication to provide lecture material without having to meet in person is advantageous, although there are some technical problems, such as insufficient internet access. ^27^^,^^28^ Lecturers highlighted the importance of using multimedia to implement online learning communication. This finding aligns with that of Palupi and Raharjo, ^29^ who reported that, in online learning communication, one alternative is to combine two online learning tools, Zoom and Google Classroom. Zoom is a virtual conferencing platform used in online learning. However, it lacks facilities for sending assignments. Google Classroom has features that allow you to send and receive assignments as well as save quotes on the Internet. Both used together will complement each other to meet all the necessities of online learning. ^29^ The lecturers in this study indicated the need to update themselves to learn about technology related to online learning communication. Shaharanee *et al.* ^30^ found that university management must address several challenges in preparing lecturers for the transition from teaching and learning to online learning. Consequently, lecturers require additional training, particularly in technical and online learning. A reliable online learning platform is required for lecturers to embark on this new experience. ^30^

## Conclusions

The findings of this study can assist lecturers in understanding online learning communication in Indonesia. These findings are crucial for university lecturers developing methods for enhancing the implementation of online learning communication.

Lecturers are important in online learning communication and can aid or hinder change. Consequently, they must be aware of how online communication affects students at the personal level. Accordingly, the necessary support should be provided to help lecturers and students cope well with online learning communication.

Moreover, the findings revealed that lecturers should engage in and create trusting interactions with their students. Providing technical support to keep lecturers and students engaged may be useful. More crucially, multimedia support and learning systems are essential for properly addressing lecturers’ tasks and learning materials.

Online learning communication is more successful when its duration is reduced. Moreover, lecturers benefit from hybrid learning methods because they can increase their self-efficacy when implementing online learning communication.

Online learning communication should not be the sole responsibility of lecturers. Students should also be a source of support. Thus, lecturers should cultivate an atmosphere that allows them and their students to enjoy online communication.

This study improves the understanding of online learning communications implemented by lecturers. As Indonesia continues to implement it, lecturers and students must respond to and actively engage in the process to ensure its success.

### Limitations

This study has a few limitations. First, it is a preliminary study that examines university lecturers’ experiences in online learning communication in Indonesia, and the challenges they face. Because this was an exploratory study, the results were inconclusive. The findings of this study can be used to build a research design or formulate a questionnaire to survey several people. Second, as some participants may have been afraid to express a negative opinion about their university, self-reported data may have lacked accuracy when examining a particular topic. Furthermore, the small number of participants (N=10) could be considered a limitation. Third, future research should explore student perspectives on online learning communication to verify these findings. This study may be replicated by integrating different research sites, sampling a larger population including students, and utilizing multiple data collection techniques. Furthermore, this study does not focus on the values, norms, and other elements of Indonesia’s sociocultural context, which may influence how lecturers view online learning communication. Instead, this research provides useful information about university lecturers’ experiences with online learning communication.

## Data Availability

Figshare: Transkrip Bahasa Indonesia New. https://doi.org/10.6084/m9.figshare.25139816. ^31^ The project contains the following underlying data:
- transkrip bahasa indonesia new.docx (Transcripts of interviews) Figshare: English Transcript New. https://doi.org/10.6084/m9.figshare.25139813. ^32^ The project contains the following underlying data:
- english transcript new.docx Figshare: Interview Guide. https://doi.org/10.6084/m9.figshare.24978213. ^33^ The project contains the following extended data:
- interview guides.docx Data are available under the terms of the Creative Commons Attribution 4.0 International license (CC-BY 4.0).

## Footnotes

^1^
Further reading: Creswell JW. A concise introduction to mixed methods research. SAGE Publications; 2014.

^2^
Further reading: Van Manen M. Phenomenology of practice: Meaning-giving methods in phenomenological research and writing. Routledge; 2016.
