# Supplementary material for: Lecturers’ experiences of online learning communication in Indonesia during the COVID-19 pandemic: A phenomenological study
Source: F1000Res. 2025 May 1;13:170. Originally published 2024 Mar 8. [Version 2] doi: 10.12688/f1000research.141212.2 (PMC12441667; doi:10.12688/f1000research.141212.2)
Supplement: Peer review history

# Reviewer report: Reviewer response for version 2

**DOI:** 10.5256/f1000research.180557.r414032

**Published:** 2025-09-16

**Authors:** I Kadek Suartama

**Affiliations:** 1 Universitas Pendidikan Ganesha, Singaraja, Bali, Indonesia

This is a strong and valuable qualitative study that addresses a timely and globally relevant topic. The use of a phenomenological approach is appropriate for capturing the rich, lived experiences of lecturers. The research is well-structured, methodologically sound, and the findings (the 10 themes) are clearly presented and well-supported by direct quotations. The revisions made from Version 1 have significantly strengthened the manuscript.

 The study makes a genuine contribution to the literature by focusing on the often-overlooked lecturer perspective within the specific socio-cultural and infrastructural context of Indonesia.

Is the work clearly and accurately presented and does it cite the current literature?

Yes

If applicable, is the statistical analysis and its interpretation appropriate?

Yes

Are all the source data underlying the results available to ensure full reproducibility?

Yes

Is the study design appropriate and is the work technically sound?

Yes

Are the conclusions drawn adequately supported by the results?

Yes

Are sufficient details of methods and analysis provided to allow replication by others?

Yes

Reviewer Expertise:

e-learning; mobile learning; ubiquitous learning

I confirm that I have read this submission and believe that I have an appropriate level of expertise to confirm that it is of an acceptable scientific standard.

## References

[1] : Learning loss: Impact of the COVID-19 pandemic on the students’ psychosocial condition. *Journal of Education and e-Learning Research* .2023;10(2) : 10.20448/jeelr.v10i2.4543 209-214 10.20448/jeelr.v10i2.4543

[2] : Instructional Design Models for Pervasive Learning Environment: Bridging Formal and Informal Learning in Collaborative Social Learning. *Education Sciences* .2024;14(12) : 10.3390/educsci14121405 10.3390/educsci14121405

[3] : Mastering knowledge construction skills through a context-aware ubiquitous learning model based on the case method and team-based projects. *International Journal of Education and Practice* .2024;12(3) : 10.18488/61.v12i3.3842 1094-1112 10.18488/61.v12i3.3842

# Reviewer report: Reviewer response for version 2

**DOI:** 10.5256/f1000research.180557.r381828

**Published:** 2025-05-24

**Authors:** Jude William Genilo

**Affiliations:** 1 University of Liberal Arts Bangladesh, Dhaka, Dhaka Division, Bangladesh

I am satisfied with the revisions made by the authors.

Is the work clearly and accurately presented and does it cite the current literature?

Yes

If applicable, is the statistical analysis and its interpretation appropriate?

Partly

Are all the source data underlying the results available to ensure full reproducibility?

Yes

Is the study design appropriate and is the work technically sound?

Yes

Are the conclusions drawn adequately supported by the results?

Yes

Are sufficient details of methods and analysis provided to allow replication by others?

Partly

Reviewer Expertise:

NA

I confirm that I have read this submission and believe that I have an appropriate level of expertise to confirm that it is of an acceptable scientific standard.

# Reviewer report: Reviewer response for version 1

**DOI:** 10.5256/f1000research.154634.r263310

**Published:** 2024-05-02

**Authors:** Jude William Genilo

**Affiliations:** 1 University of Liberal Arts Bangladesh, Dhaka, Dhaka Division, Bangladesh

In general, the study is important as it provides the experiences of lecturers regarding online courses during the pandemic era. The study was conducted satisfactorily. However, there are a few aspects that can be done to improve the research as follows:

1. The researcher can provide a more comprehensive discussion on the study’s context. For one, they can narrate the pandemic situation government actions to curb the pandemic, government actions (including support given like providing internet access, mobile phones, surveys, online platforms) on preventing the spread in schools and universities. Another, they can talk about internet access and social media use in the country during the study period. Also, they can give a brief introduction and description about UPN Veteran, including prior experience and training provided on hybrid or online learning, policies towards online learning, faculty number, policies during pandemic era regarding class attendance, etc.
2. The objectives can be made more specific. For example, what are the categories of experiences that they would like lecturers to share about – technology, pedagogy, adopting content, student performance, student interaction, co-learning experiences, etc.
3. There should be greater detail regarding the background of lecturers such as age, number of years teaching, training received on online learning, social media and internet use, class size, type of class taught whether theoretical, lab or field based, etc.
4. Regarding related literature, there have been several studies written about teachers’ experiences during the pandemic – both quantitative and qualitative. More can be cited by the researchers. They can go to the open access HEED journal.
5. Regarding the results, there should be a discussion about the typical online class experience of lecturers. How do they start classes? How do they prepare? Do they check attendance? What are their ground rules? How did they adjust their course outline to fit the new pedagogy? Did they change the ways they evaluate students? How did they take account about the students’ mental health situation? Did they collaborate with student affairs office? How was their reporting system to their chair or dean?
6. The researchers can also ask the lecturers about the lesson they learned during the pandemic and whether there are still some practices that they carry over to the post-pandemic situation.
7. To allow replication by others, the researchers may provide a description of the guide questions. Also, the researchers should state whether there were other artifacts that were looked into like PowerPoint presentations and class recordings of the sessions.

Is the work clearly and accurately presented and does it cite the current literature?

Yes

If applicable, is the statistical analysis and its interpretation appropriate?

Partly

Are all the source data underlying the results available to ensure full reproducibility?

Yes

Is the study design appropriate and is the work technically sound?

Yes

Are the conclusions drawn adequately supported by the results?

Yes

Are sufficient details of methods and analysis provided to allow replication by others?

Partly

Reviewer Expertise:

NA

I confirm that I have read this submission and believe that I have an appropriate level of expertise to confirm that it is of an acceptable scientific standard, however I have significant reservations, as outlined above.

# Response

**Published:** 2025-04-11

**Authors:** Lukman Waluyo

**Affiliations:** UPN Veteran Jakarta, Indonesia

**The researcher can provide a more comprehensive discussion on the study’s context. For one, they can narrate the pandemic situation government actions to curb the pandemic, government actions (including support given like providing internet access, mobile phones, surveys, online platforms) on preventing the spread in schools and universities. Another, they can talk about internet access and social media use in the country during the study period. Also, they can give a brief introduction and description about UPN Veteran, including prior experience and training provided on hybrid or online learning, policies towards online learning, faculty number, policies during pandemic era regarding class attendance, etc.:**

**Author Response: **During the COVID-19 pandemic, the Indonesian government implemented several measures to curb the virus's spread, particularly within educational institutions. In March 2020, President Joko Widodo called for social distancing, leading to the closure of schools and universities nationwide. This prompted a swift transition to online learning, with the Ministry of Education and Culture collaborating with the Ministry of Communication and Information Technology and state-owned enterprises to enhance telecommunications infrastructure and support digital education platforms. Despite these efforts, challenges such as limited internet access and inadequate devices persisted, particularly in remote areas

 Internet usage in Indonesia surged during the pandemic, with social media penetration increasing from 59% in January 2020 to 68.9% by early 2022, and average daily usage nearing four hours. This rise in digital engagement highlighted the growing reliance on online platforms for information and communication during the crisis

**The objectives can be made more specific. For example, what are the categories of experiences that they would like lecturers to share about – technology, pedagogy, adopting content, student performance, student interaction, co-learning experiences, etc:**

**Author Response: **

- **To explore lecturers’ experiences with technology usage**: Investigate how lecturers interact with various digital platforms and tools (e.g., Zoom, Google Meet, LMS), including challenges related to access, training, and integration into teaching practices.
- **To examine pedagogical adaptations in online learning environments**: Understand how lecturers adjust their teaching methods and instructional strategies to suit virtual or hybrid settings.
- **To identify approaches to content adaptation and delivery:** Explore how lecturers modify or redesign learning materials (videos, readings, presentations, etc.) to be suitable for asynchronous or synchronous learning formats.
- **To analyze lecturers’ perspectives on student performance and engagement**: Assess how online learning has influenced students’ academic performance, motivation, and focus, from the viewpoint of the lecturers.

**To investigate student–lecturer interaction in online settings**: Examine the nature and quality of interaction during online classes, including feedback, participation, and communication challenges.

# Reviewer report: Reviewer response for version 1

**DOI:** 10.5256/f1000research.154634.r263313

**Published:** 2024-04-17

**Authors:** Sherwyn P Morreale

**Affiliations:** 1 University of Colorado, Colorado Springs, Colorado, USA

This qualitative phenomenological study represents a useful contribution to an area of inquiry that is contemporarily critical to any instructor or administrator with an interest in enhancing online education in higher education, locally or globally. The introduction and literature review point to a two-part rationale for the study’s research questions:  (1) a gap in understanding online learning and communication from lecturers’ perspectives, and (2) the need to conduct such a study in the Indonesian culture. The interesting qualitative phenomenological method is carefully explained to ensure replicability, which could occur comparatively in other cultures. The data analysis section of the method is interesting and well described for the reader. Most important, the ten themes described in the results section are clearly described and illustrated by including direct quotations from the study’s participating lecturers.

 These strengths aside, this review now provides some recommendations intended to enhance the writing style and presentation of this study and its findings. Some of these recommendations are molar  and relate to the overall writing while others are molecular and more specific.

**Molar Recommendations**

1. Consider clarifying terms that may be varyingly used by readers in various cultures and countries. Describe what is meant by “lecturers.” Are these full-time faculty, or part-time instructors/teachers, and are they fully responsible for teaching all aspects of a given course or courses? The term lecturer could mean a teacher who  only speaks to students, which is lectures  only, one-way communication. Also describe  the types of online delivery of the courses presented by the ten lecturers in the study. Are the courses fully and solely delivered online, or are they  hybrid, that is, part online and part in-seat/traditional delivery? Clarify these and any other essential terms that may be misunderstood, early in manuscript, perhaps toward the start of the methods section.
2. Regarding writing style: Throughout the manuscript, sometimes only one sentence  constitutes a paragraph. Instead, cluster ideas that clearly relate to one another in longer paragraphs that start with a lead-in line; for example, in the literature review: ” Research to date has provided insight into students ‘ viewpoints  of their experiences with online teaching and learning.”  Then describe the types of  studies  already included in the literature review about that topic. But don’t include in the literature review any opinions that are not supported by scholarly research, published or presented literature.
3. Perhaps the discussion and conclusions sections could  be somewhat more expansive.  Two possible ways to be more expansive would be: in the discussion, harken back to and answer the study’s three research questions;  answer each of the three with several sentences of discussion. Also, add Table 2 to the discussion. List  the ten themes presented in the results and include a 1-2 sentence of each theme. That table could go at the end of the results.

**Molecular Recommendations **

1. This sentence in the introduction may suggest that there is a dearth of research about communication education in online education. While there is a need for this and other such  studies, considerable studies have been published in the U.S. and internationally (see for example 13 studies published in: *Frontiers in Communication: Instructional Communication Competence and Instructor Social Presence.*
2. In the methods section, provide an explanatory sentence of Table1, perhaps phrased like this.

Table 1

 The ten participating lecturers included  one lecturer from each of the following ten areas of study at the university.

  

1. At the end of the third paragraph of the data collection section, change the word ponder’ s to study’s.
2. Be consistent with capitalization throughout, for example: Capitalize Internet when it’s used as a noun. Also proof section headings and subtitles for consistency of capitalization.
3. In the third paragraph of the Results section, (Lack of Engagement), add a lecturer statement from the results so this theme is the same as the other themes.
4. In the Results section:

Change the  subtitle from Hybrid learning based on the subject to: Variability in learning based on the subject

 Change the subtitle Maximizing Efficiency of Online Larning Communication to: Maximizing efficiency and effectiveness of online learning communication  

 In the Results section, subtitled Learning method, change A participant believes, to One lecturer believes…

Is the work clearly and accurately presented and does it cite the current literature?

Partly

If applicable, is the statistical analysis and its interpretation appropriate?

Yes

Are all the source data underlying the results available to ensure full reproducibility?

Yes

Is the study design appropriate and is the work technically sound?

Yes

Are the conclusions drawn adequately supported by the results?

Yes

Are sufficient details of methods and analysis provided to allow replication by others?

Yes

Reviewer Expertise:

instructional communication, communication education, online education, communication competence, public speaking

I confirm that I have read this submission and believe that I have an appropriate level of expertise to confirm that it is of an acceptable scientific standard, however I have significant reservations, as outlined above.

# Response

**Published:** 2025-04-11

**Authors:** Lukman Waluyo

**Affiliations:** UPN Veteran Jakarta, Indonesia

**Consider clarifying terms that may be varyingly used by readers in various cultures and countries. Describe what is meant by “lecturers.” Are these full-time faculty, or part-time instructors/teachers, and are they fully responsible for teaching all aspects of a given course or courses? The term lecturer could mean a teacher who  only speaks to students, which is lectures  only, one-way communication. Also describe  the types of online delivery of the courses presented by the ten lecturers in the study. Are the courses fully and solely delivered online, or are they  hybrid, that is, part online and part in-seat/traditional delivery? Clarify these and any other essential terms that may be misunderstood, early in manuscript, perhaps toward the start of the methods section:**

 Response - The term “lecturer” in this study refers to a faculty member whose primary responsibility is teaching, who works full-time and who is fully responsible for all aspects of course instruction, including preparing materials, delivering lectures, and assessing students’ work. A lecturer does not simply give lectures; they often engage students through discussions, projects, and other interactive methods. 

**In the discussion, harken back to and answer the study’s three research questions; answer each of the three with several sentences of discussion. Also, add Table 2 to the discussion. List  the ten themes presented in the results and include a 1-2 sentence of each theme. That table could go at the end of the results:**

 Response - The author team have updated the table

**At the end of the third paragraph of the data collection section, change the word ponder’ s to study’s**. --> done

**Be consistent with capitalization throughout, for example: Capitalize Internet when it’s used as a noun. Also proof section headings and subtitles for consistency of capitalization. **--> done

**Change the  subtitle from Hybrid learning based on the subject to: Variability in learning based on the subject** --> done

**Change the subtitle Maximizing Efficiency of Online Larning Communication to: Maximizing efficiency and effectiveness of online learning communication  **--> done

**In the Results section, subtitled Learning method, change A participant believes, to One lecturer believes…** --> done
